# Supplementary material for: The Oral Administration of Sanguisorba officinalis Extract Improves Physical Performance through LDHA Modulation
Source: Molecules. 2021 Mar 12;26(6):1579. doi: 10.3390/molecules26061579 (PMC7998416; doi:10.3390/molecules26061579)
Supplement: Supplementary file 1 [file molecules-26-01579-s001.pdf]

molecules

Supplementary Materials

The Oral Administration of *Sanguisorba officinalis* Extract Improves Physical Performance through LDHA Modulation

Jung Ho Han [1,2], MinJeong Kim [3], Hee-Jin Choi [1,2], Jung Sook Jin [2], Syng-Ook Lee [4], Sung-Jin Bae [2], Dongryeol Ryu [3,*] and Ki-Tae Ha [1,2,*]

1 Department of Korean Medical Science, School of Korean Medicine, Pusan National University, Yangsan, Gyeongnam 50612, Korea; hanjh1013@pusan.ac.kr (J.H.H.); choih@musc.edu (H.-J.C.)
2 Healthy Aging Korean Medical Research Center, Pusan National University, Yangsan, Gyeongnam 50612, Korea; jinpaldook@hanmail.net (J.S.J.); dr.nowornever@pusan.ac.kr (S.-J.B.)
3 Department of Molecular Cell Biology, Biomedical institute for Convergence at SKKU (BICS), School of Medicine, Sungkyunkwan University (SKKU), Suwon, Gyeonggi-do 16419, Korea; alswjd0105@skku.edu
4 Department of Food Science and Technology, Keimyung University, Daegu 42601, Korea; synglee@kmu.ac.kr
* Correspondence: freefall@skku.edu (D.R.); hagis@pusan.ac.kr (K.-T.H.)

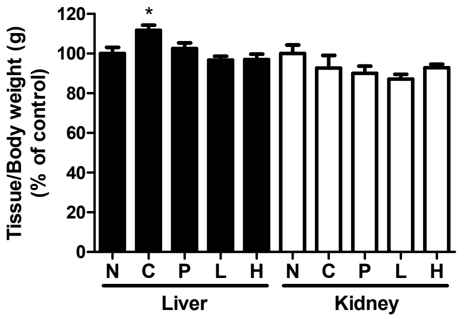

Figure S1. Measurement of drug toxicity. The weight of the liver and kidney was measured. The results are shown as mean ± SEM. * $p < 0.05$ compared to each N group.

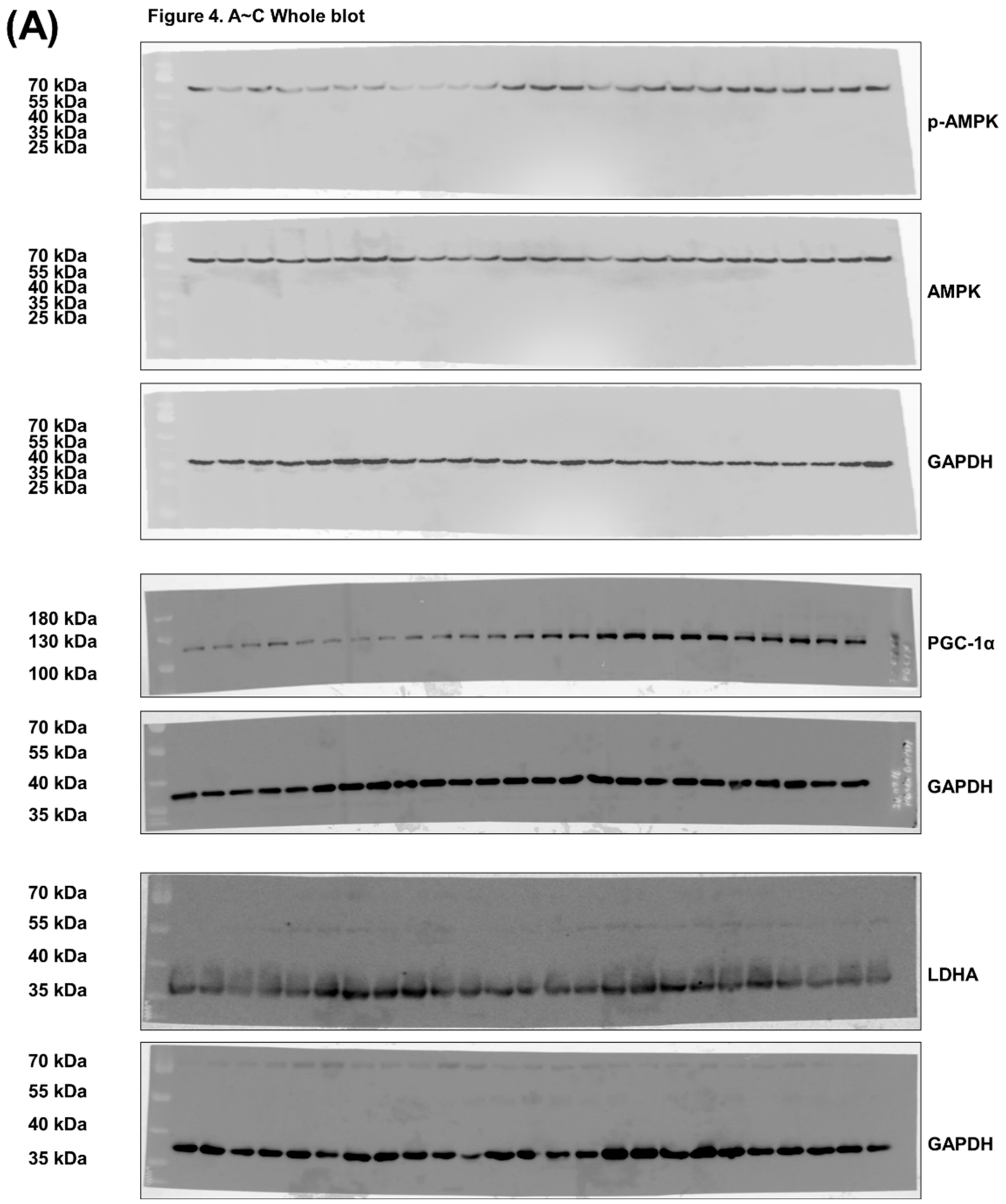
(A)
Figure 4. A~C Whole blot
70 kDa
55 kDa
40 kDa
35 kDa
25 kDa
p-AMPK
70 kDa
55 kDa
40 kDa
35 kDa
25 kDa
AMPK
70 kDa
55 kDa
40 kDa
35 kDa
25 kDa
GAPDH
180 kDa
130 kDa
100 kDa
PGC-1α
70 kDa
55 kDa
40 kDa
35 kDa
GAPDH
70 kDa
55 kDa
40 kDa
35 kDa
LDHA
70 kDa
55 kDa
40 kDa
35 kDa
GAPDH

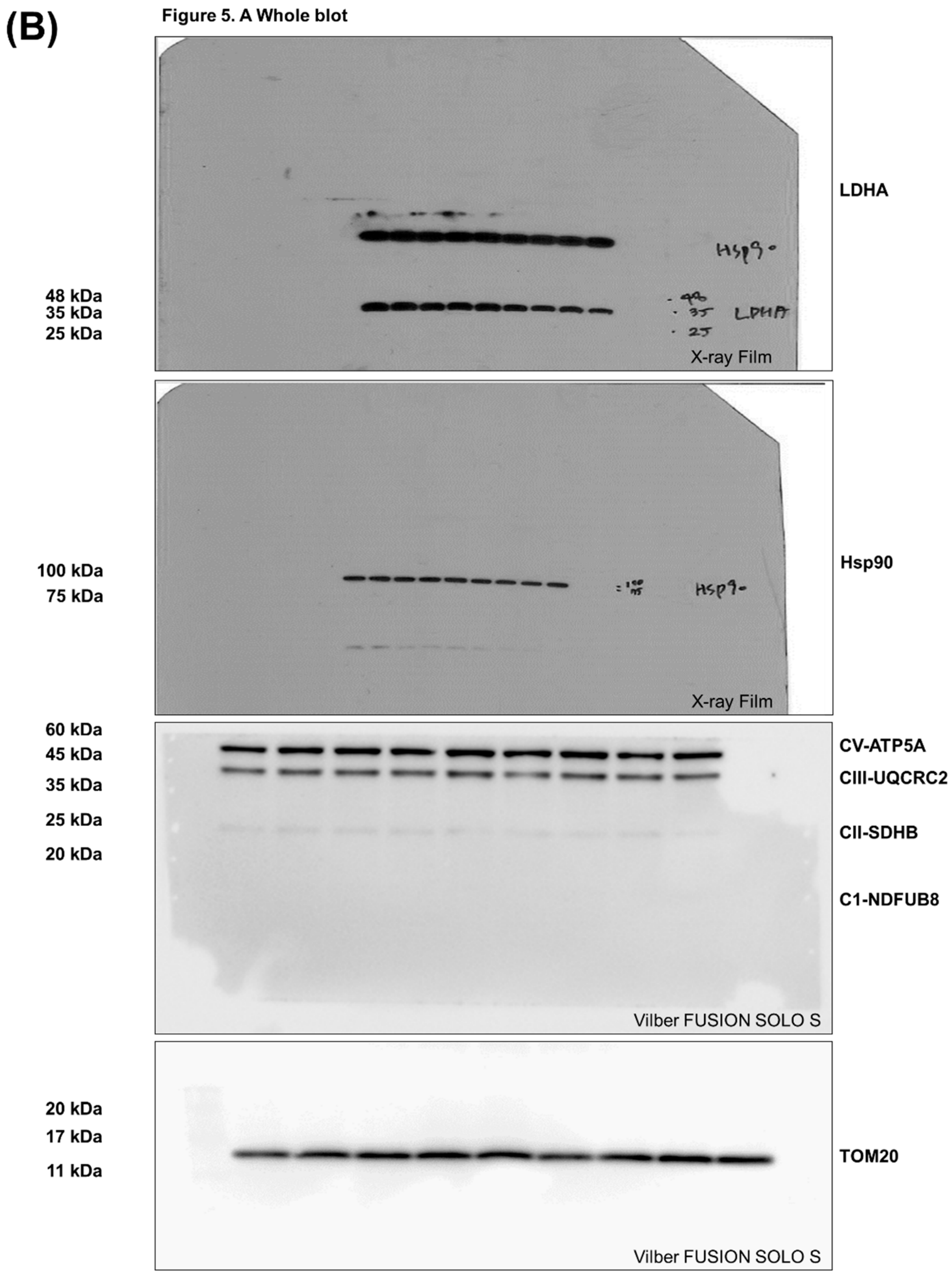

Figure S2. The whole blot of Western blot analysis. This figure shows the raw data of Western blot assay in this study.
